# Stigma Toward People With Mental Illness Among Romanian Psychiatry Trainees: A Mixed-Methods Study

**DOI:** 10.7759/cureus.90941

**Published:** 2025-08-25

**Authors:** Elena A Manescu, Claire Henderson, Ciprian R Paroiu, Alex C Boaca, Horia Marchean, Adriana Mihai

**Affiliations:** 1 Department of Psychiatry, George Emil Palade University of Medicine, Pharmacy, Science, and Technology of Târgu Mureș, Târgu Mureș, ROU; 2 Health Services and Population Research, David Goldberg Centre, Institute of Psychiatry, Psychology and Neuroscience, King's College London, London, GBR

**Keywords:** mental health professionals, mhpsi, mixed-methods, psychiatry trainees, qualitative, stigma

## Abstract

Background

Stigma associated with mental illness is a critical barrier to effective care, and emerging evidence suggests that stigmatizing attitudes exist even among mental health professionals. In Romania, cultural and systemic factors may exacerbate this issue, yet research examining stigma within this group is limited. This mixed-methods study investigated the attitudes and behaviors of Romanian psychiatry trainees toward individuals with mental illness, their knowledge of mental health stigma, and their perspectives on its causes and potential anti-stigma strategies.

Methods

Qualitative data were collected through individual interviews, and a quantitative assessment was conducted using the Mental Health Provider Stigma Inventory (MHPSI), with 20 participating trainees. Qualitative data were analyzed using thematic analysis, and mean differences were assessed for the total and subscale scores of the MHPSI.

Results

Quantitative analysis revealed moderate overall levels of stigma, with no significant differences in total or subscale scores across academic years. Statistically significant differences between males and females were observed for the total score (T(17) = 2.19, p = 0.04) and the “coworker influence” subscale (T(18) = 3.13, p = 0.002), with males scoring higher than females. Qualitative findings indicated that manifestations of stigma, such as diagnostic overshadowing and avoidance, were readily recognized among healthcare professionals. However, only a minority of psychiatry trainees acknowledged stigma in their own clinical practice, particularly in the form of judgmental attitudes and a lack of empathy. Key factors contributing to stigma included insufficient stigma education, institutional culture, and burnout. Trainees highlighted the need for targeted interventions to reduce stigma in clinical interactions.

Conclusions

These findings underscore that both individual and systemic factors contribute to stigma among psychiatry trainees. Addressing this issue requires comprehensive, context-specific anti-stigma interventions integrated into medical curricula and residency programs, with a focus on stigma training and reflective practice.

## Introduction

Mental health stigma, defined by Link and Phelan [1] as negative attitudes, beliefs, and behaviors toward individuals with mental illness, has gained significant attention as a critical barrier to effective care and recovery [2,3]. While public stigma is widely recognized, emerging research indicates that stigma is also prevalent among mental health professionals, including psychiatrists [3-5].

Stigmatizing attitudes within the professional community often manifest through negative stereotypes, biased clinical practices, and subtle discriminatory behaviors [3-5]. Psychiatrists may display specific stigmatizing attitudes and behaviors that adversely affect patient care. For example, some may adopt a paternalistic approach by undervaluing patient autonomy, assuming that patients are incapable of making informed decisions regarding their treatment [4-7]. Additionally, diagnostic overshadowing is a frequent concern, in which physical complaints presented by patients with mental illness are prematurely attributed solely to their psychiatric condition, potentially leading to underdiagnosis or mismanagement of comorbid physical health issues [3,8,9]. Perceived negative affective reactions and perceived social distance from mental health providers contribute to the overall internalized stigma and disempowerment of patients with mental illness [10]. Moreover, expressing pessimistic views regarding recovery, using derogatory labels (e.g., “noncompliant” or “manipulative”), and subtle nonverbal cues, such as avoiding eye contact, further reinforce negative stereotypes [6,8,9,11].

Research on mental health stigma in Romania, as in other low- and middle-income countries, remains limited and has focused primarily on public stigma, leaving a significant gap in our understanding of stigma among mental health professionals [12,13]. To our knowledge, there is limited research on stigma among healthcare professionals and no scientific evidence regarding stigma among mental health professionals in Romania [14], reflecting the broader global scarcity of evidence on stigma within this professional group. Limited resources, lack of prioritization of mental health care, and gaps in specialized training [14] indicate the presence of structural stigma and are likely to contribute to the persistence of interpersonal stigma among mental health professionals.

Given their potential to serve as catalysts for change, research in this population is essential for developing effective, tailored interventions to reduce stigma and improve mental health care outcomes [15-17]. Therefore, this study aimed to examine the attitudes and behaviors of Romanian psychiatry trainees toward individuals with mental illness, as well as their perspectives on the contributing factors to mental health stigma and potential strategies for its reduction. It was hypothesized that psychiatry trainees would present moderate to high levels of stigma toward individuals with mental illness, influenced by both structural and interpersonal factors, and that they would identify a need for enhanced training and systemic support to effectively address stigma.

## Materials and methods

Design

A mixed-methods design was employed to comprehensively assess stigma as a complex, context-sensitive issue, integrating qualitative and quantitative approaches in line with methodological borrowing [18]. Semi-structured interviews explored trainees’ perceptions and experiences, uncovering implicit attitudes, cultural narratives, and training gaps often missed by quantitative tools. These insights informed the quantitative component, which provided measurable, generalizable data.

Setting and participants

The study was conducted between August and October 2024 in a psychiatry clinic located in Târgu Mureș, Romania. The clinic provides both short- and long-term psychiatric services for a wide range of mental disorders. All psychiatry residents working at the hospital (n = 51) were invited to participate using a purposive sampling technique. Recruitment was facilitated through an announcement by the academic supervisor, supplemented by printed posters and informational leaflets displayed in the hospital.

Inclusion criteria required participants to be enrolled in the psychiatry residency program and actively working at the hospital during the data collection period. Psychiatry residents from first through fourth years participated: three from the first year (15%), five from the second (25%), five from the third (25%), and seven from the fourth year (35%). The psychiatry residency program in Romania spans five years; fifth-year residents were not represented in the sample, likely due to their focus on preparing for the national specialty certification examination.

Participants provided informed consent after receiving detailed information about the study. No financial compensation was offered, and participants were not in a subordinate or professional relationship with the principal investigator, who conducted all one-on-one interviews to ensure confidentiality and reduce bias.

Data collection and analysis

Qualitative Data

Semi-structured interviews included open-ended questions based on a previous literature review [14]. The principal researcher had prior experience conducting qualitative interviews, ensuring methodological rigor and sensitivity. Interviews lasted 10-20 minutes, after which participants completed the MHPSI.

The interview questions covered three main topics: common knowledge of mental health stigma, attitudes and behaviors of health and mental health professionals toward psychiatric patients, and potential strategies to reduce stigma and discrimination in the healthcare system. The topic guide was used flexibly and revised as the analysis progressed. Interviews were audio-recorded with participants’ permission and transcribed for analysis.

Transcripts were analyzed using Braun and Clarke’s six-phase framework for thematic analysis at a semantic level [19]. Due to the small sample size, qualitative data analysis software was not used; instead, a manual coding approach was adopted. Two members of the research team conducted the analysis independently. Coding began deductively, applying a preliminary framework based on the interview guide and an initial reading of the transcripts, including pre-identified categories related to stigma-related attitudes and behaviors. Subsequently, an inductive analysis allowed for the emergence of unanticipated themes. Codes were iteratively refined, and categories adjusted as new patterns emerged. To enhance reliability, the two researchers compared coding, resolved discrepancies, and refined theme definitions through discussion and consensus, ensuring the integrity and richness of participants’ perspectives.

Quantitative Data

The original, untranslated version of the Mental Health Provider Stigma Inventory (MHPSI) was used [20]. The MHPSI is a 24-item instrument divided into three domains: attitudes toward mental health clients (nine items), observable actions reflecting stigmatizing attitudes (eight items), and perceived influence of coworker attitudes and behaviors on individual provider views (seven items). Items are rated on a 7-point Likert scale (1 = completely disagree; 7 = completely agree), with one item reverse-scored. The MHPSI has demonstrated good reliability and validity, with a global stratified alpha of 0.95.

Descriptive statistics were calculated for total and subscale scores, with variables presented as means ± SDs and medians. Female participants were expected to record lower scores [3], and a decrease in scores was anticipated across academic year groups. Normality and equal variance assumptions were met; therefore, an independent samples t-test was used to compare male and female groups, and a one-way ANOVA assessed differences across academic years. Statistical significance was set at p < 0.05. All analyses were conducted using GraphPad Prism (version 10, GraphPad Software Inc., San Diego, CA, USA).

Ethical considerations

The study was approved by the ethics committee of the George Emil Palade University of Medicine, Pharmacy, Science, and Technology of Târgu Mureș (approval 3333) and the ethics committee of the hospital where the study was conducted (approval 6298).

## Results

Approximately 40% (N = 20) of the psychiatry residents working at the hospital participated in the study. Of these, 20% (N = 4) were male and 80% (N = 16) were female, reflecting the overall male-to-female ratio of psychiatrists in the hospital. The mean age of participants was 28.2 years (SD = 1.36).

Qualitative results

Thematic analysis identified 20 codes that clustered into four main themes: stigma in healthcare providers, stigma in mental health professionals, possible causes of stigma among psychiatrists, and strategies for reducing stigma. The results of the thematic analysis, along with participants’ perspectives, are presented in Table 1.

**Table 1:** Results of thematic analysis

Codes	Themes	Participants’ perspectives	Prevalence across interviews (%)
Discrimination, avoidance, prejudice, insufficient care, diagnostic overshadowing, dismissal	Stigma among healthcare providers	Premature discharge of patients with mental illness from general medical wards due to staff concerns about potential behavioral deterioration. Neglect of medical symptoms in psychiatric patients, or misattribution to their mental illness (diagnostic overshadowing). Delayed interdepartmental consultations for psychiatric inpatients despite clear clinical indications.	100
Labelling, paternalism, prejudice, avoidance, disregard, disrespect, judgment, low empathy, patients most stigmatized	Stigma among mental health professionals	Limited communication about diagnosis and treatment options; lack of involvement in shared decision-making. Dismissive, rushed communication; referring to patients primarily by their diagnosis. Nursing staff displayed a notable lack of empathy and respect, with occasional reports of hostile or aggressive behavior. Certain patients, particularly those with frequent hospitalizations or specific personality traits, tended to be avoided by psychiatrists. Patients with borderline personality disorder were frequently stigmatized and avoided, perceived as difficult and unpredictable. Similarly, those with histrionic traits or substance use disorders were often harshly judged and socially devalued.	30
Poor stigma education/awareness, burnout, institutional culture	Causes of stigma in mental health professionals	Significant resource constraints, including shortages of medication, insufficient psychological support during hospitalization, and a lack of free post-discharge resources for patients. Attitudes and behaviors toward patients were influenced by subconscious modelling of supervisors and colleagues. The topic of stigma toward psychiatric patients is rarely addressed in formal medical education or residency programs.	75
Mandatory training during psychiatric rotations in medical school, training during residency programs in psychiatry, post-university courses, psychotherapy	Ways of reducing stigmatizing attitudes and discriminatory behaviors	Training in addressing patients without stigmatization should be an essential and mandatory part of medical education to ensure it is taken seriously and applied consistently. Although psychotherapy is recommended, its financial burden falls on the trainee; psychotherapeutic approaches emphasizing the patient-physician relationship were perceived as particularly beneficial.	100

Stigma Among Healthcare Providers

Discussions on the stigmatization of psychiatric patients primarily focused on the attitudes and behaviors of healthcare professionals across multiple medical specialties. All participants reported witnessing at least one instance in which a psychiatric patient experienced avoidance, substandard care, or neglect by healthcare providers.

Stigma Among Mental Health Professionals

Only a minority of participants recognized stigmatizing attitudes and behaviors among psychiatrists and other mental health professionals. Reported behaviors included avoidance, judgmental attitudes toward patients’ character, and a general lack of empathy. Participants acknowledged the frequent use of diagnostic labels when referring to patients. One participant noted that stigma also manifests in the failure to involve patients in therapeutic decisions and in providing insufficient or no explanations regarding their mental disorder, treatment options, and prognosis.

Patients perceived to experience the highest levels of stigma from psychiatrists were those with a history of frequent hospitalizations, substance abuse, or certain personality disorders, such as borderline, histrionic, or antisocial traits. These patients were often avoided or treated with minimal empathy.

Causes of Stigma in Mental Health Professionals

Participants identified the main causes of stigma as lack of education and poor awareness of stigma’s manifestations and impact, institutional culture, and burnout. Criticizing or complaining about patients was described as a common aspect of institutional culture. Burnout was primarily associated with frustration toward the mental health care system and working environment, rather than excessive workload. Notably, none of the participants reported receiving formal training on stigma.

Ways to Reduce Stigma

Participants generally recommended mandatory anti-stigma modules during psychiatry rotations in medical school, training during residency programs, and postgraduate courses. Some participants also suggested that psychotherapy should be considered essential for psychiatrists.

Quantitative results

Table 2 presents the means, SDs, medians, minimums, and maximums for the MHPSI total and subscale scores. The mean total score indicated low to moderate stigma. No significant differences were observed in total or subscale scores across academic years (Table 3). A comparison between males and females revealed a significant difference for the total score (T(17) = 2.19, p = 0.04) and the “coworker influence” subscale (T(18) = 3.13, p = 0.002), with males scoring higher than females (Table 4).

**Table 2:** Descriptive statistics of the MHPSI and subscales scores MHPSI: Mental Health Provider Stigma Inventory

MHPSI	Mean (SD)	Median	Minimum	Maximum
Total score	58.3 (14.14)	62	35	75
Subscales				
Attitudes	22.31 (4.29)	22	15	30
Behaviors	17.94 (6.64)	19	8	28
Coworker influence	16.63 (7.33)	7.21	7	31

**Table 3:** Results of ANOVA between academic years in total and subscales scores Data are presented as means (SD) and coefficient of variance (F). Statistical significance was set at p < 0.05. MHPSI: Mental Health Provider Stigma Inventory

Academic year/MHPSI	I	II	III	IV	F	p
Total score	62 (8.19)	53.4 (17.4)	67.6 (5.59)	53.57 (16.25)	1.31	0.3
Attitudes	26.33 (3.51)	19 (3.32)	23.6 (3.36)	22.29 (4.27)	2.66	0.08
Behaviors	17 (6.24)	16.8 (9.88)	19.2 (5.16)	17.85 (5.92)	0.11	0.95
Coworker influence	17.33 (5.77)	14 (7.28)	21.6 (7.33)	14 (6.92)	1.41	0.27

**Table 4:** Differences between males and females in total and subscales scores Data are presented as means (SD) and the statistical coefficient of difference between means (T). Statistical significance was set at p < 0.05; bold values indicate significant results. MHPSI: Mental Health Provider Stigma Inventory

MHPSI	Males	Females	T (df)	p
Total score	70 (3.74)	54.2 (14)	2.19 (17)	0.04
Attitudes	22 (2.44)	22.5 (4.58)	0.2 (18)	0.4
behaviors	21.25 (6.65)	16.93 (6.38)	1.2 (18)	0.1
Coworker influence	24.75 (5.9)	14.31 (5.97)	3.13 (18)	0.002

## Discussion

Our findings indicate that stigma is present both in the broader healthcare system and within the field of mental health. Although participants had not received formal education on stigma associated with mental illness, they recognized stigmatizing attitudes and behaviors, more often in others than in themselves, and more frequently among general healthcare professionals than among mental health professionals.

Stigma among health and mental health professionals

Participants more readily identified stigma among general healthcare providers than among mental health professionals. Manifestations included premature discharges, inadequate care, and avoidant or dismissive behaviors, often attributed to general medical staff, particularly regarding diagnostic overshadowing. Although fewer participants acknowledged stigma within mental health settings, some reported avoidance, judgmental attitudes, and a lack of empathy, especially the failure to involve patients in treatment decisions, behaviors consistent with prior findings on exclusionary and paternalistic practices in psychiatric care [3,6,7,21].

Such practices undermine therapeutic collaboration, patient autonomy, and adherence, potentially worsening outcomes [10]. Shared decision-making and clinician awareness of stigma’s impact have been identified as key strategies to improve care [17,22,23]. Participants also noted that nurses sometimes exhibited the least empathy, reinforcing evidence that stigma may be embedded in institutional culture [24]. Certain patient groups were disproportionately labeled as “difficult” or “a disgrace,” reflecting harmful stereotyping that can lead to poorer outcomes and further stigmatization, a pattern supported by recent literature [4,17].

Factors associated with stigma in psychiatrists

Our findings suggest that stigma among mental health professionals is driven by insufficient education, institutional culture, and burnout rooted in systemic challenges. Participants described a workplace norm in which complaining about patients was normalized, reflecting how institutional ethos and role modelling shape attitudes. This environment perpetuates stigma as both a cause and a consequence of systemic dysfunction. Burnout, characterized as stemming from resource shortages rather than workload, was associated with reduced empathy and increased stigma, suggesting that addressing systemic resource issues could simultaneously reduce provider burnout and improve attitudes toward patients [25,26].

Participants also highlighted the lack of formal anti-stigma education and consistently recommended its inclusion in medical and residency training. Recent studies support the efficacy of such interventions in reducing stigma and fostering a more empathetic, informed clinical practice [15-17]. Some participants suggested that regular psychotherapeutic support for psychiatrists could buffer against burnout and stigma. Evidence indicates that reflective practices, such as Balint groups and mindfulness, can effectively reduce burnout and promote more empathetic, less stigmatizing behaviors toward patients [27].

Quantitative insights and gender differences

Quantitative findings from this study provide insights into the levels and dynamics of stigma among psychiatry residents as measured by the MHPSI. Overall, total MHPSI scores and subscale scores indicated moderate levels of stigma, consistent with previous research in mental health settings [4,17].

No significant differences in total or subscale scores were observed across academic years, suggesting that the current training, which lacks anti-stigma modules, does little to shift these perceptions over time. Attitudes and behaviors associated with stigma may become established early in training and persist throughout residency. Similar findings in the literature indicate that stigma-related beliefs can become entrenched during early professional socialization, with limited change over the course of medical education or residency [3,15,28].

Regarding gender differences, male psychiatry trainees reported higher overall levels of stigma and higher scores on the coworker influence subscale, consistent with prior literature [3]. Interestingly, the attitudes and behavior subscales did not differ significantly by gender, suggesting that men’s higher overall scores may reflect contextual and interpersonal dynamics, such as peer influence and workplace culture, rather than strongly held explicit beliefs. This aligns with evidence that trained mental health professionals can unconsciously hold or express stigmatizing views, particularly when such behaviors are modelled or normalized in their professional context [22]. Corrigan and colleagues [29] emphasize that stigma is often embedded within organizational cultures, where certain stereotypes or distancing behaviors toward patients are implicitly accepted or reinforced.

The observed gender difference is particularly relevant given the local workforce composition. At our institution, the mental health workforce is female-dominated (83%), a pattern mirrored among psychiatry trainees (73% female vs. 27% male). National data from Romania indicate a slightly less pronounced female majority (approximately 70% female vs. 30% male) [30]. This local imbalance may amplify social pressures: male trainees may feel compelled either to resist dominant norms to preserve masculine identity or to align with perceived stigmatizing attitudes among a subset of colleagues to secure social belonging and professional credibility [31].

This pattern also reflects broader literature on gender and stigma, which suggests that men are more likely to conform to traditional masculine norms, such as emotional control, independence, and toughness, that are often incongruent with empathy or openness to mental health issues [32,33]. In environments where compassion and engagement with individuals with mental illness are not overtly valued or modelled by peers, as indicated by our qualitative findings, male providers may adopt more stigmatizing postures to assert professional legitimacy or avoid social disapproval.

It is important to note, however, that women may also be susceptible to workplace peer pressure. Research indicates that social and interpersonal dynamics in healthcare settings can have differential impacts on gender, with women often showing greater sensitivity to nuances in workplace culture and peer influences [34,35].

Overall, these findings suggest that local gender distribution may play a critical role in shaping workplace influences on stigma, highlighting the importance of considering institutional demographics when interpreting results. Future research should investigate whether similar patterns occur in settings with a more balanced gender distribution and explore targeted interventions designed to reduce stigma in mental health care by addressing these specific contextual factors.

Strengths and limitations

This study is the first to examine Romanian psychiatric trainees’ attitudes toward individuals with mental illness and their perceptions of stigma within professional settings, and it is among the few studies on this topic conducted globally. Using a mixed-methods design, the study captured both personal and perceived attitudes, helping to mitigate social desirability bias. However, its exploratory nature, small sample from a single hospital, and limited participation (40%) constrain generalizability. While findings may not fully reflect broader trends, substantial differences are unlikely given the standardized national training, which lacks stigma education.

Future studies should recruit larger, more diverse samples to enhance generalizability and enable regional or institutional comparisons. Longitudinal and cross-national designs could further clarify how trainees’ attitudes toward mental illness evolve over time and vary across cultural and training contexts. Experimental research is also warranted to assess the impact of targeted stigma-reduction interventions, such as contact-based education or reflective practice, within psychiatric training programs. In-depth qualitative and mixed-method approaches, potentially incorporating social network analysis, could elucidate how organizational culture, peer influence, and gender dynamics interact to shape stigma in mental health settings.

## Conclusions

The multifaceted phenomenon of stigma requires comprehensive strategies that address not only individual attitudes but also the broader institutional culture that perpetuates stigmatizing practices. Interventions aimed at managing stigmatizing attitudes, such as stereotyping, labelling, and rejection, as well as challenging the normalization of harmful practices, may be beneficial.

Preliminary findings indicate that stigma levels persist across different stages of training in the absence of formal anti-stigma education. Therefore, early and continuous integration of anti-stigma content into medical education and psychiatric residency programs appears promising. Interventions incorporating reflective practices on coworker influence and institutional dynamics, such as Balint groups, may be particularly effective. Such efforts can help foster more inclusive and responsive mental health care environments. Future research should examine longitudinal changes in stigma across different stages of professional development and rigorously evaluate the effectiveness of tailored interventions, particularly those targeting both institutional and interpersonal dimensions of stigma.
